# Adaptation Aftereffects in the Perception of Radiological Images

**DOI:** 10.1371/journal.pone.0076175

**Published:** 2013-10-11

**Authors:** Elysse Kompaniez, Craig K. Abbey, John M. Boone, Michael A. Webster

**Affiliations:** 1 Department of Psychology, University of Nevada, Reno, Nevada, United States of America; 2 Department of Psychological and Brain Sciences, University of California Santa Barbara, Santa Barbara, California, United States of America; 3 Department of Biomedical Engineering, University of California Davis, Davis, California, United States of America; 4 Department of Radiology, Medical Center, University of California Davis, Sacramento, California, United States of America; University College London, United Kingdom

## Abstract

Radiologists must classify and interpret medical images on the basis of visual inspection. We examined how the perception of radiological scans might be affected by common processes of adaptation in the visual system. Adaptation selectively adjusts sensitivity to the properties of the stimulus in current view, inducing an aftereffect in the appearance of stimuli viewed subsequently. These perceptual changes have been found to affect many visual attributes, but whether they are relevant to medical image perception is not well understood. To examine this we tested whether aftereffects could be generated by the characteristic spatial structure of radiological scans, and whether this could bias their appearance along dimensions that are routinely used to classify them. Measurements were focused on the effects of adaptation to images of normal mammograms, and were tested in observers who were not radiologists. Tissue density in mammograms is evaluated visually and ranges from "dense" to "fatty." Arrays of images varying in intermediate levels between these categories were created by blending dense and fatty images with different weights. Observers first adapted by viewing image samples of dense or fatty tissue, and then judged the appearance of the intermediate images by using a texture matching task. This revealed pronounced perceptual aftereffects – prior exposure to dense images caused an intermediate image to appear more fatty and vice versa. Moreover, the appearance of the adapting images themselves changed with prolonged viewing, so that they became less distinctive as textures. These aftereffects could not be accounted for by the contrast differences or power spectra of the images, and instead tended to follow from the phase spectrum. Our results suggest that observers can selectively adapt to the properties of radiological images, and that this selectivity could strongly impact the perceived textural characteristics of the images.

## Introduction

Despite advances in assistive technologies such as computer-aided detection algorithms, the evaluation and interpretation of medical images still relies ultimately on visual inspection by humans, and thus remains fundamentally constrained by the perceptual and cognitive capacities of the observer [1]. For example, approximately 30-40% of false negative diagnoses in clinical radiology are thought to result from perceptual errors [2-4]. A wide variety of studies have explored the sensory processes that impact visual judgments about medical images. These include analyses of the factors affecting the detection and discrimination of patterns [5,6], the properties of visual search and salience [7-9], and the role of perceptual learning and expertise [10]. Common to each of these has been the attempt to understand how standard visual processes and constraints are manifest in the context of the specific stimulus statistics characterizing medical images [11,12]. In this study, we explore the role of a further well-known perceptual process that is intimately linked to the visual structure defining the image – visual adaptation. The sensitivity and response properties of the visual system are constantly adjusted through adaptation to match visual coding to the attributes of the stimuli we are currently viewing [13-15]. Here we ask whether these adjustments occur for attributes of medical images in ways that could influence how such images are perceived and classified.

Adaptation is pervasive in sensory systems and can lead to dramatic changes in perception, as illustrated by many classic perceptual aftereffects [13,16]. For example, the perceived shape, color, or movement of a stimulus can be strongly biased by even a brief prior exposure to a different stimulus (e.g. so that a gray field appears greenish after adapting to a red field, and a static display appears to drift upward after adapting to downward motion). Work over the last two decades has shown that these aftereffects extend to high-level aspects of visual coding and can thus affect even complex and seemingly abstract judgments about images, such as the characteristics of a face [17] or the perceived layout of a scene [18]. They have also been shown to be readily invoked by the types of patterns we typically encounter in natural viewing [19,20]. That is, the visual environment is itself a potent stimulus for adaptation, and characteristic changes in the environment can lead to consistent and characteristic changes in the state of adaptation of the observer.

We asked how observers might adapt to the novel “visual environment” presented by medical images. These images provide an ideal context for probing the processes and consequences of visual adaptation because they have well defined properties that differ from the statistics of natural images. For instance, the power spectra of medical images is typically steeper than natural spectra [5]. This reduction in amplitude at high spatial frequencies is similar in some respects to image blur, which is known to induce rapid and strong adaptation [21]. Moreover, radiologists can spend hours at a time inspecting the images, and this long-term scrutiny seems well suited to generating robust adaptation. Thus it is plausible to expect that the simple act of viewing medical images could induce strong and specific states of adaptation that could influence their appearance. However, to our knowledge the potential impact of adaptation on the perception and performance with medical images has not been previously considered. 

**Figure 1:**
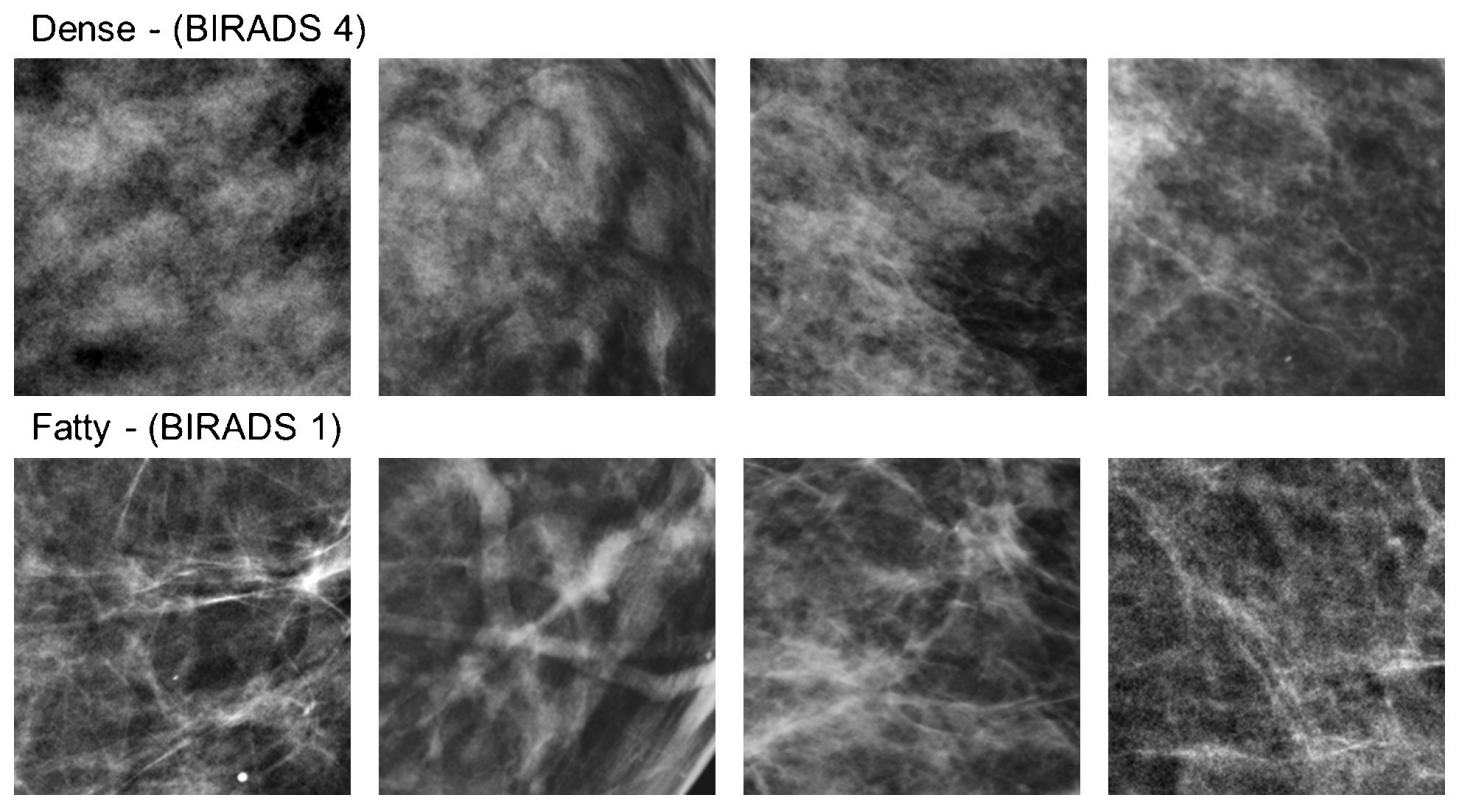
Examples of image samples from mammograms classified as dense or fatty. The 4 top and bottom pairs show the specific image pairs used to construct the test arrays.

To explore this, we investigate x-ray mammogram images on a standard diagnostic stimulus dimension that radiologists rely on to assess them. The BI-RADS Density classification system is used to rate the breast tissue on a scale of fatty to dense, and is based on visual inspection of the textural properties of the mammogram [22]. A rating of fatty implies that the tissue is composed of almost entirely fat, and is characterized by a striated appearance of the image (Figure 1). A rating of dense instead signifies the presence of fibroglandular tissue, and corresponds to an image that appears cloudy. Dense tissue lowers the sensitivity of the mammogram [23] because it obscures lesion detection [24]. Moreover, these classifications are important because they are related to the potential prevalence of cancer. For example tissue density has been correlated with 4-6 times greater incidence of breast cancer [25], and women who were administered Tamoxifen and showed a reduction in density revealed a 63% decrease in the risk of breast cancer [24]. Thus whether an image is perceived as dense or fatty has important implications for patient health and for subsequent diagnostic tests. In the current study we examined whether the textural differences that distinguish dense or fatty images could be biased by prior adaptation to dense or fatty mammograms. 

As an initial test of this question, we have focused on demonstrating the existence and magnitude of visual adaptation to these textural properties of mammography images, using tasks to assess adaptation that are straightforward and based on standard experimental paradigms in visual perception. Our focus was also on understanding adaptation to special properties of the image rather than within special populations of observers, and for this reason, we used subjects that are trained for each task, but do not have medical training. Radiologists are highly trained to make an absolute judgment to classify an individual image in terms of how dense or fatty it appears. For our untrained observers this would not be possible, so we instead adopted a procedure in which they were only required to make a relative judgment about which of two presented images appeared more dense or fatty. This allowed us to assess both the extent and form of any possible aftereffects. Our results suggest that there can in fact be profound and rapid aftereffects to the structural properties of medical images, and these have potential implications both for medical image perception and more generally for characterizing how perceptual judgments within unique visual environments might be impacted by routine processes of sensory adaptation.

## Materials and Methods

### Observers

Six observers with corrected-to-normal acuity participated in different subsets of experiments. The observers included authors EK and MW (labeled S4 and S6 in figures) and 4 students who were naïve to the purpose of the study. Participation was with written informed consent and followed protocols approved by the University of Nevada, Reno Social Behavioral Institutional Review Board (Office of human research protection).

### Apparatus and stimuli

Stimuli were presented on a calibrated and gamma-corrected Sony 500 PS monitor controlled by a Cambridge Research Systems VSG graphics card. The images were displayed on a gray background on the monitor with the same chromaticity and mean luminance (~37 cd/m^2^).

**Figure 2:**
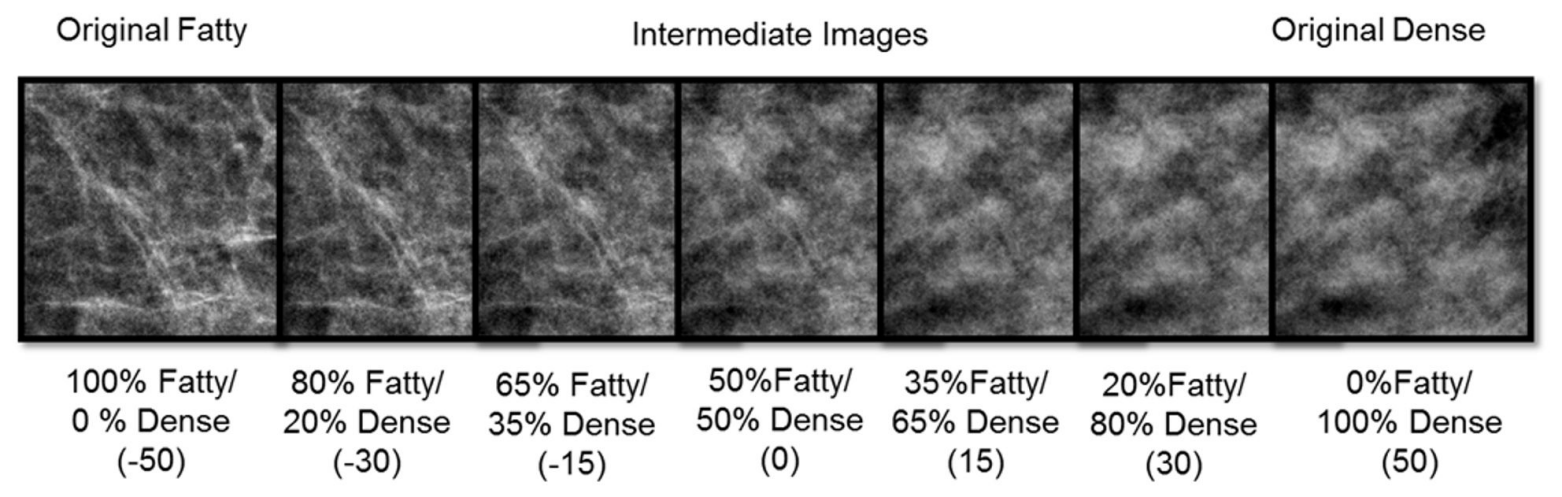
An example of the test stimulus array formed by different weighted averages of a dense and fatty image.

The stimuli consisted of randomly selected sections taken from a database of normal mammograms [26] previously classified with BI-RADS Density scores of “fatty” vs. “dense” (values of 1 or 4), again corresponding to differences in the relative quantities of fat vs. fibroglandular tissue. The sections corresponded to 256 by 256 pixels in the original 2560 by 3328 images, and were constrained to be fully within the breast region of the image. The 8-bit pixel values were rescaled so that the average luminance (37 cd/m^2^) and rms contrast (.38) was constant across all images. Sets of these images taken from mammograms classified as dense or fatty served as the adapting stimuli. For test stimuli, we further created an array of images that varied in finely graded steps between the dense and fatty originals. This was done by averaging the pixel levels from a pair of fatty and dense images, varying the relative weighting to form 101 images spanning the range. An image of +50 corresponded to the original dense image and an image of -50 corresponded to the original fatty image, while image 0 corresponded to an equal mixture of the two (Figure 2). As with the adapting stimuli all test images had the same mean luminance and contrast. 

### Procedure

Stimuli were viewed binocularly in a darkened room from 124 cm. At this distance the images subtended 4 deg (~1 arcmin per pixel), and were displayed within fields centered 2.2 deg on the left or right of a central black fixation cross. Specific experiments varied in whether the adaptation was to a single image or sets of images and whether the adapting images were shown in one field or both, as described below. In all cases, observers initially adapted for 60 sec to fatty or dense images, with the adapting field counterbalanced between the left and right sides. Baseline measurements were also taken following adaptation to a uniform field. The adapting stimuli filled the 4-deg displayed window (which was 228 x 228 pixels), but their position within it was randomly jittered every 100 ms over a range of +16 pixels to avoid local light adaptation**.**

Following adaptation, a probe image was presented in the adapting field(s), and had a level chosen from different points along the fatty-dense array. A matching image with variable level was shown in the opposite field. The probe and match images were displayed simultaneously for 250 ms, and were preceded and followed by a 100 ms gray field. The participants made a 2-alternative response to indicate whether the match image appeared “too fatty” or “too dense” relative to the probe. (The stimulus directions corresponding to these responses could be learned quickly from whether the chosen response caused the two images to converge or diverge in appearance.) Subsequent test stimuli were shown interleaved with 4 sec periods of readaptation, with the array level of the match stimulus varied in a staircase (i.e. an array step toward the dense image if the response was “too fatty” or vice versa). The experiment terminated after 10 reversals of the staircase, and the level at which the two test images appeared to match (i.e. when the two alternative responses were equally likely) was estimated from the mean of the final 6 reversals. Observers made 4 or more repeated measurements for each adapt and test condition in counterbalanced order, with a different pair of dense/fatty exemplars on each run. The reported results are based on the average of these settings. 

## Results

**Figure 3:**
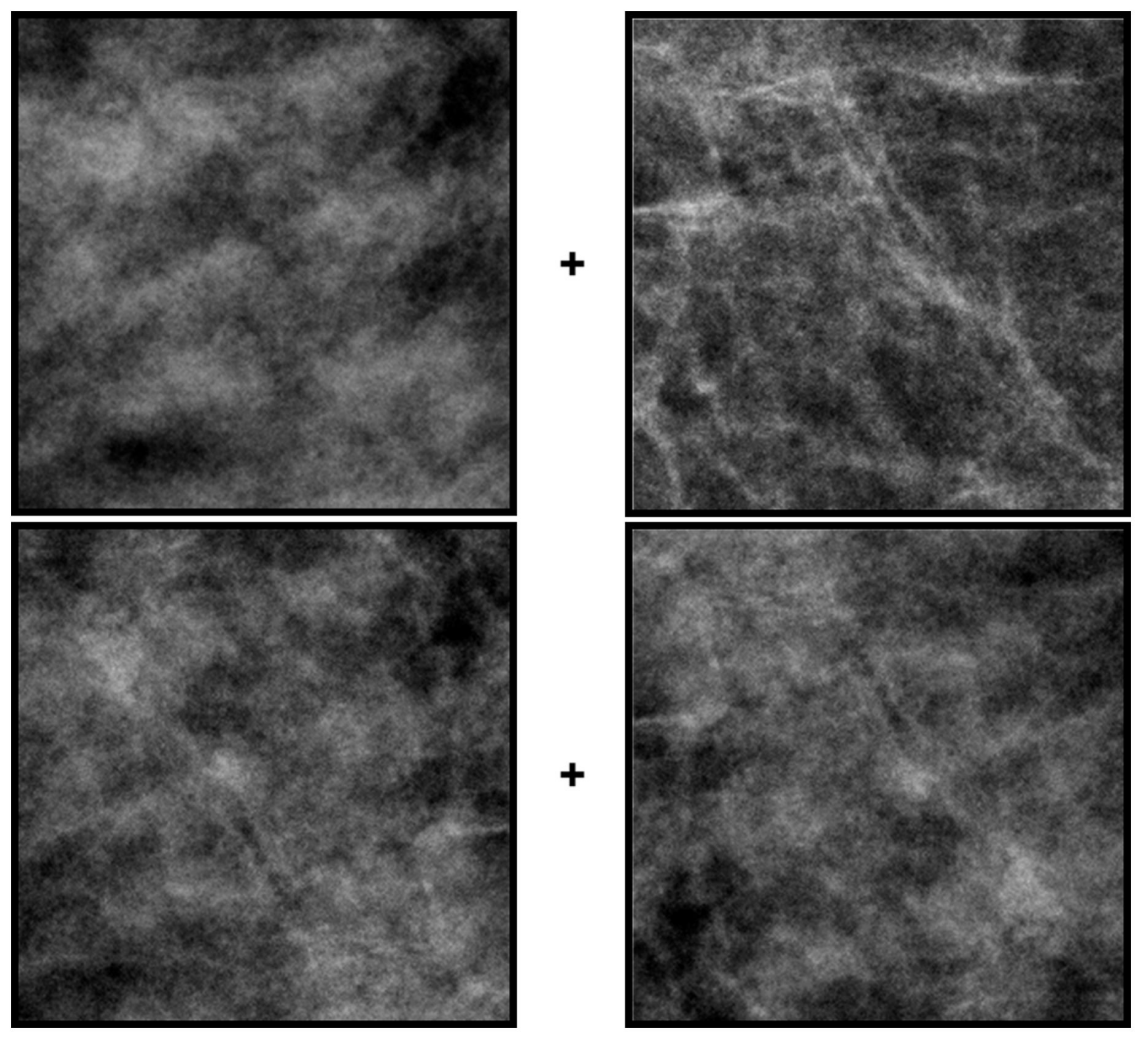
A demonstration of aftereffects induced by adaptation to dense or fatty images. Stare at the cross between the top pair of dense (left) and fatty (right) images for 30 sec and then quickly shift gaze to the cross between the bottom images. These are an average of the two top images, and are physically the same on the left and right (but mirror reversed). However, after adapting the right image should briefly appear more dense.

Figure 3 provides a simple demonstration of the basic textural aftereffects induced by the dense and fatty images. (see Video S1.) The top pair of images is again two sections from original mammograms classified as dense (left) or fatty (right). The bottom pair is the same on the left and right and was formed by averaging the two top images. Fixating the cross between the top images for several seconds should induce adaptation to the dense or fatty texture within each field. If fixation is quickly shifted to the lower cross, then the physically identical pair may briefly appear different – the right image should appear more dense than the left. Consistent with most adaptation aftereffects, the perceptual change is a “negative aftereffect” because the test image appears less like the adapting image, and results because the adaptation selectively reduces sensitivity to the adapting image [16].

To quantify the perceptual shifts, in the first experiment we adapted to both a dense and fatty image in the separate fields as in Figure 3, and then asked observers to adjust the pair of test images with the staircase procedure until they appeared the same. This turned out to be an easy task for observers and also had the advantage that the rms contrast between the two fields remained constant, so that the aftereffects could not be attributed to a simple aftereffect of apparent contrast. The procedure also had the advantage that it provided a sensitive probe of any perceptual shift, since the two test fields should be biased by adaptation in opposite ways, amplifying the appearance difference. To measure this difference, the levels of the two test images were yoked to vary symmetrically around the 50% average, and observers judged whether the right image was more dense or more fatty. That is, a “too fatty” response caused the next displayed pair to be more dense on the right but more fatty on the left.

**Figure 4:**
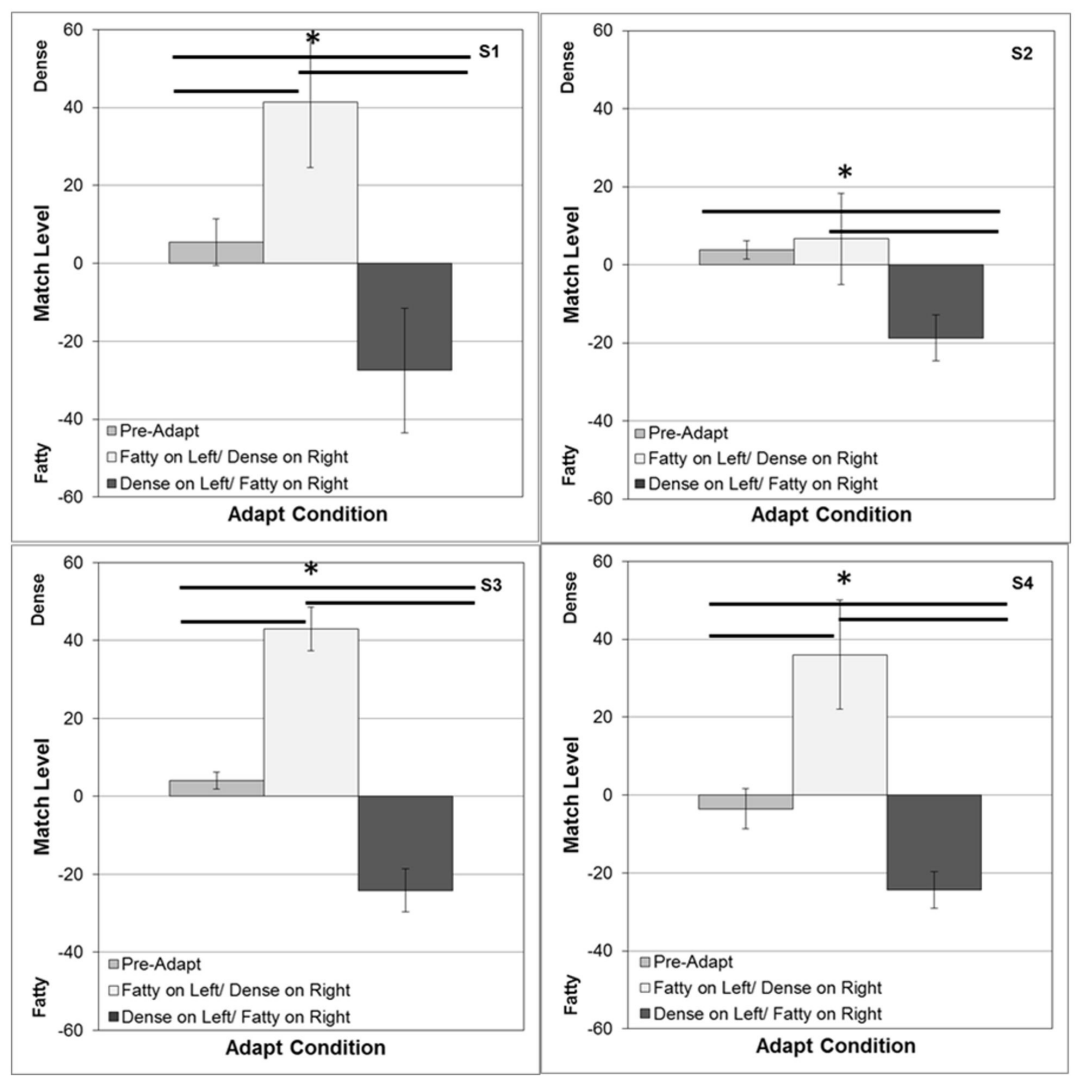
Aftereffects measured with opposite adapting images (dense vs. fatty) displayed in the left and right fields. Each panel shows for one observer the array level of the image on the right that appeared to match the image on the left. Test images were yoked so that when the image was, for example, 40 on the right it was paired with a -40 image on the left. Bars show the mean settings +1 standard error, when there were no adapting images (left), when the dense image was on the right and fatty on the left (middle), or when the positions were reversed (right). Horizontal lines indicate significant differences in the settings for the 3 conditions.

Figure 4 shows the average settings for 4 observers. Under neutral adaptation (to a gray screen), the settings approximate a physical match. However, after adapting to fatty images on the left (and dense on the right), the test image on the right appeared too fatty, so that the perceived point of equality was strongly shifted to denser images on the right (and more fatty on the left). Not surprisingly, strong complementary aftereffects also occurred when the locations of the two adaptors were switched. Thus in both cases physical differences had to be introduced between the two test images in order to null out the perceptual differences resulting from the adaptation. These aftereffects were significant both relative to neutral adaptation and between the two alternate locations of the adaptors. (For the mean settings across observers, neutral vs. dense t(6)=-3.37, p = .008; neutral vs. fatty t(6)=9.65, p < .0001; dense vs. fatty t(6) = 6.40, p = .0003).

**Figure 5:**
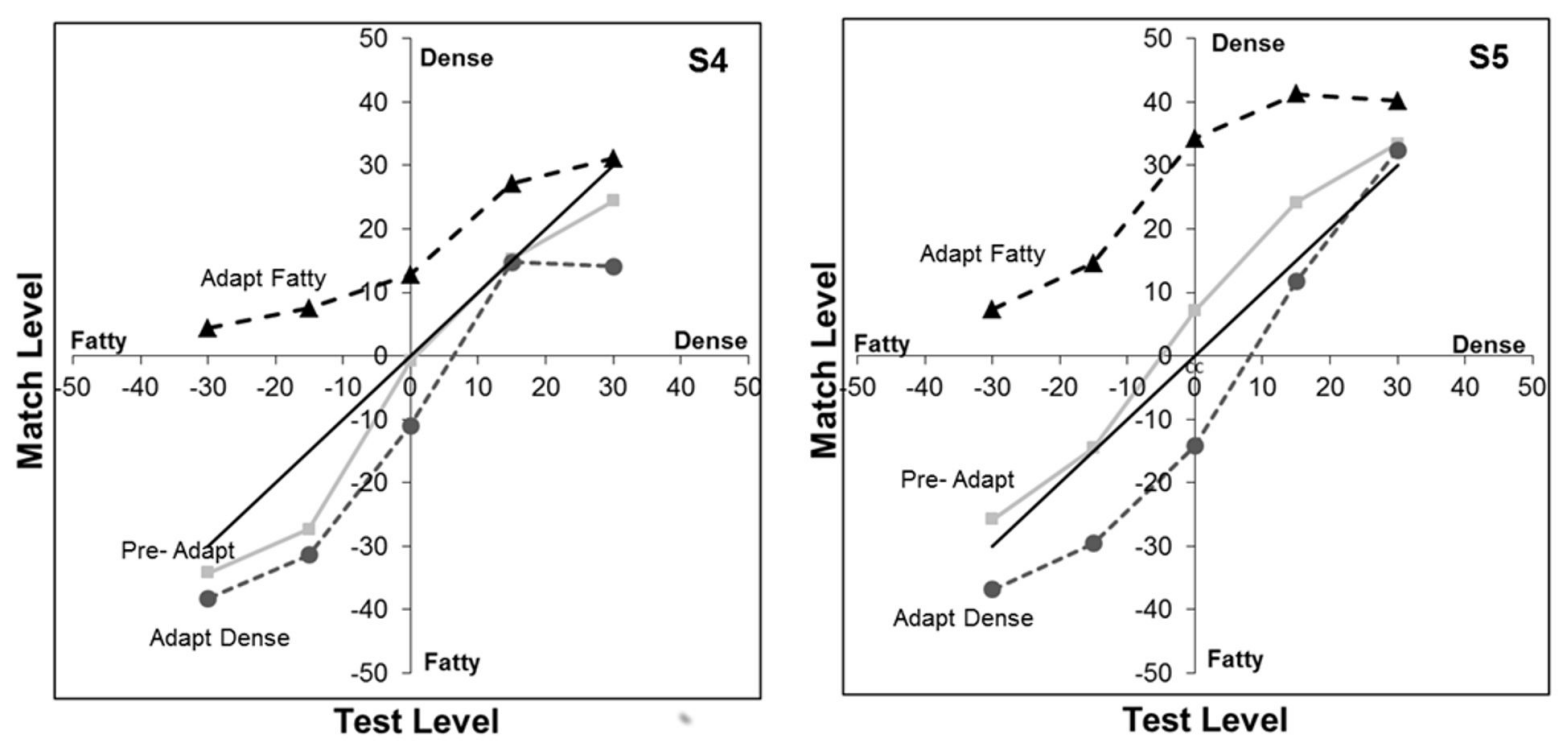
Aftereffects on different levels of blended fatty vs. **dense textures**. Curves show the stimulus levels that matched different levels of the test probe before adapting (squares) or after adapting to the fatty (triangles) or dense image (circles). The solid diagonal line corresponds to a physical match. The 2 panels are for 2 observers.

To further assess the form of the aftereffect, in the second experiment we modified the task so that the adaptation was presented only on the left or right, and so that the probe shown in the adapting field had a constant level. This allowed us to more directly characterize how the appearance of different probe levels were altered by the dense or fatty adaptor since the matching stimulus was no longer also altered by the adaptation (to the extent that the adaptation is specific to the retinal location stimulated, consistent with the opposite aftereffects seen in Figure 4). Figure 5 shows the settings for two observers who matched intermediate probe levels ranging from -30 (80% fatty) to +30 (80% dense). Again, the neutral adapt settings roughly follow the physical match (positive diagonal). After adapting to the fatty images, all of the probe levels appear more dense and thus were equated with a matching level that was physically more dense. Conversely, dense adaptors instead shifted the appearance toward more fatty images, though the magnitude of the aftereffect in the dense case appears weaker. Settings for both observers revealed a highly significant main effect of the adapt condition on the image appearance (S1 F(2,45)=29.5, p<.001; S2 F(2,45)=58.3, p<.001). However, they differed in whether there was an interaction between the adapt condition and test level (S1 F(8,45)=1.94, p=.077; S2 F(8, 45), p=.011).

**Figure 6:**
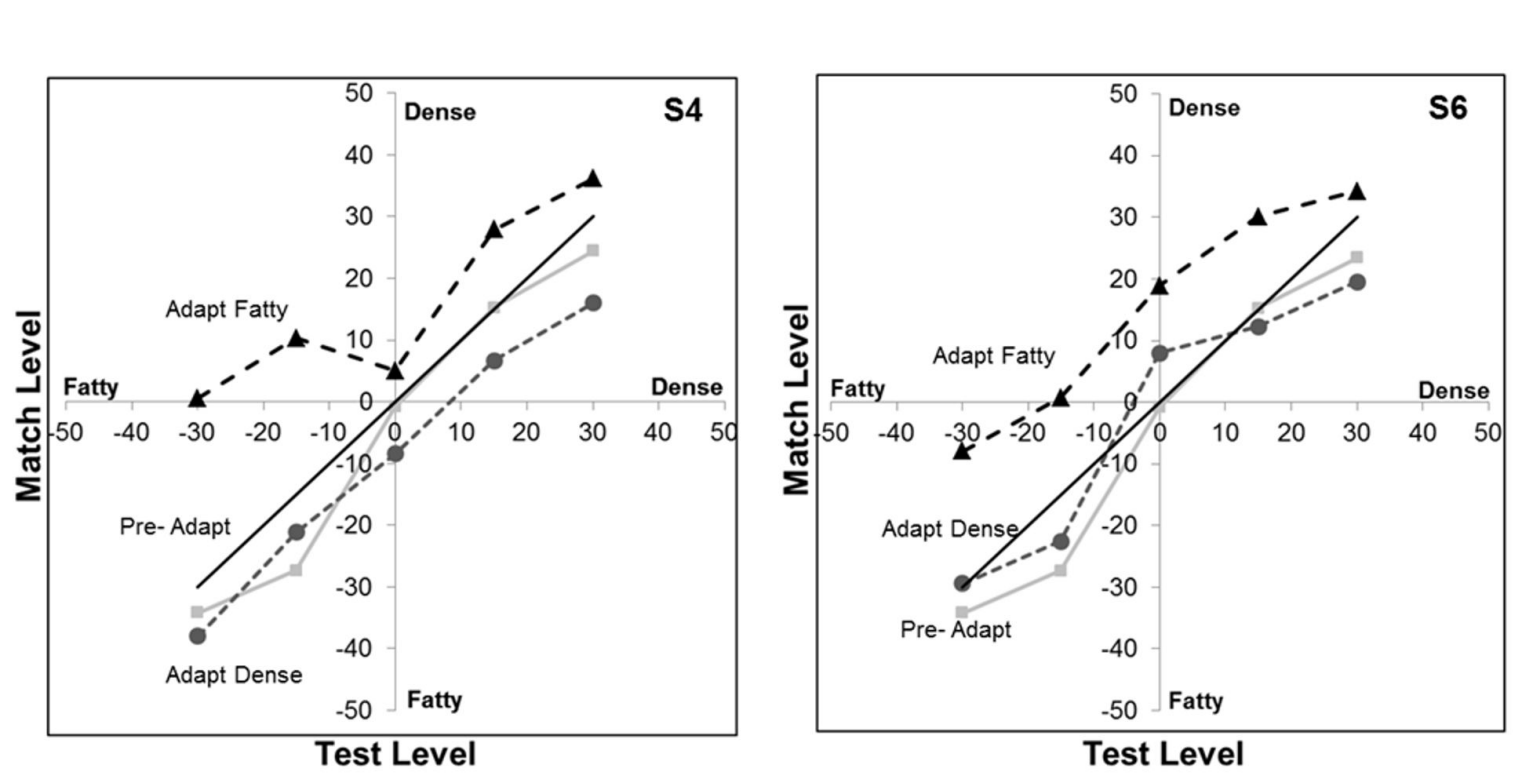
Transfer of the aftereffects across different images. Adaptation was to a sequence of dense or fatty images that differed from the images used to construct the test arrays. Curves show the stimulus levels that matched different levels of the test probe before adapting (squares) or after adapting to the fatty (triangles) or dense image (circles). The solid diagonal line corresponds to a physical match. The 2 panels are for 2 observers.

As noted in Methods, the position of the adapt image was jittered during presentation to prevent aftereffects from local light adaptation to the bright and dark regions of the image. However, we next evaluated whether these aftereffects reflected adaptation to the specific pattern of the individual mammogram, or whether they could be also be induced by the dense and fatty textural attributes of the image regardless of which images were carrying those attributes. For this, aftereffects were again assessed for a range of probe levels, but the single adapting image was replaced with a series of dense or fatty exemplars which were different from the image pair used to construct the test array. Settings for two observers are shown in Figure 6, and are similar to the results found for the single adapting images. There is again a significant main effect of the adapt condition (S1 F(2, 45)=21.7, p<.001; S2 F(2, 45)=10.7, p<.001) that is stronger for the fatty adaptors and did not interact with the probe level (S1 F(8, 45)=1.53, p=.18; S2 F(8, 45)=.35, p=.94). The similar effects in this case suggest that adaptation can in fact adjust to the actual textural properties defining the dense or fatty images, and that these aftereffects can transfer from one mammogram image to another.

**Figure 7:**
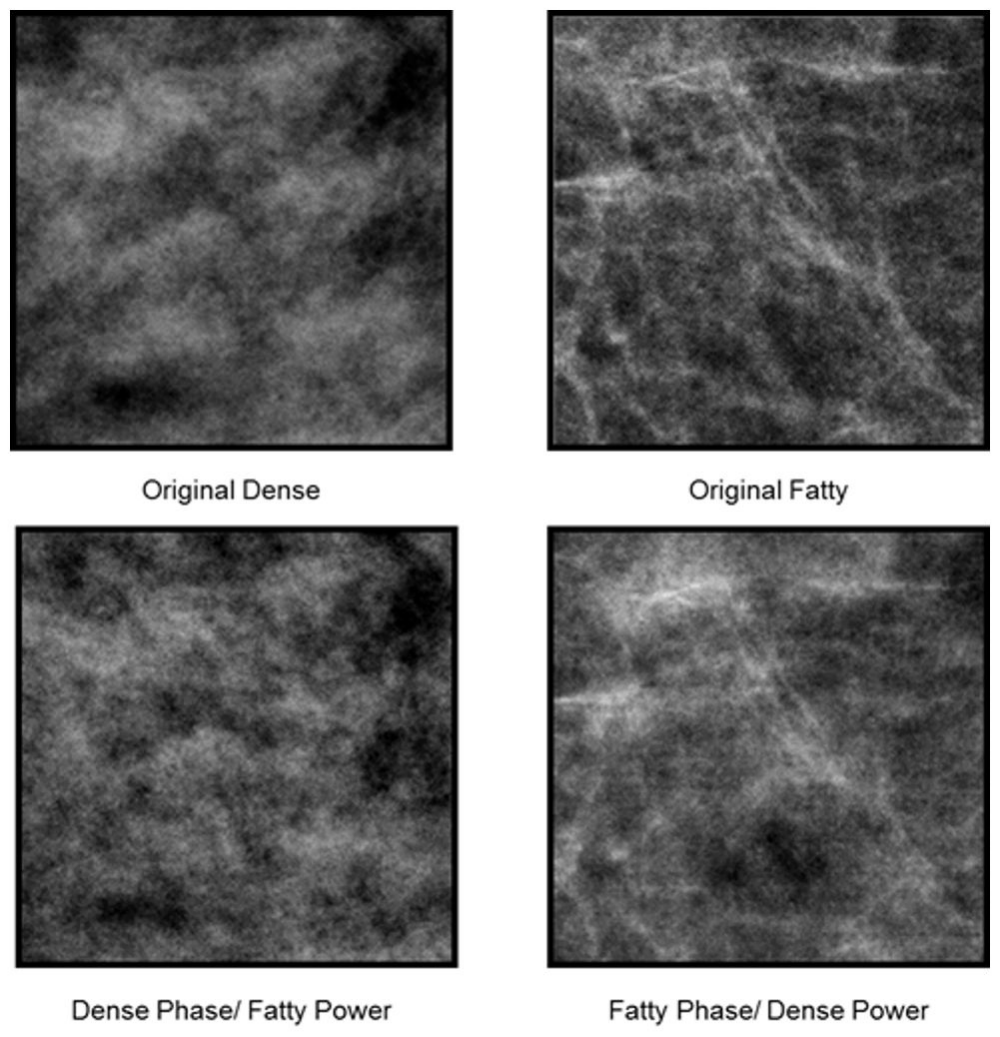
Examples of image pairs before or after swapping the power spectra. The top pair shows the original images, while the bottom pair has the power spectrum from the image above it but the phase spectrum of the second original.

**Figure 8:**
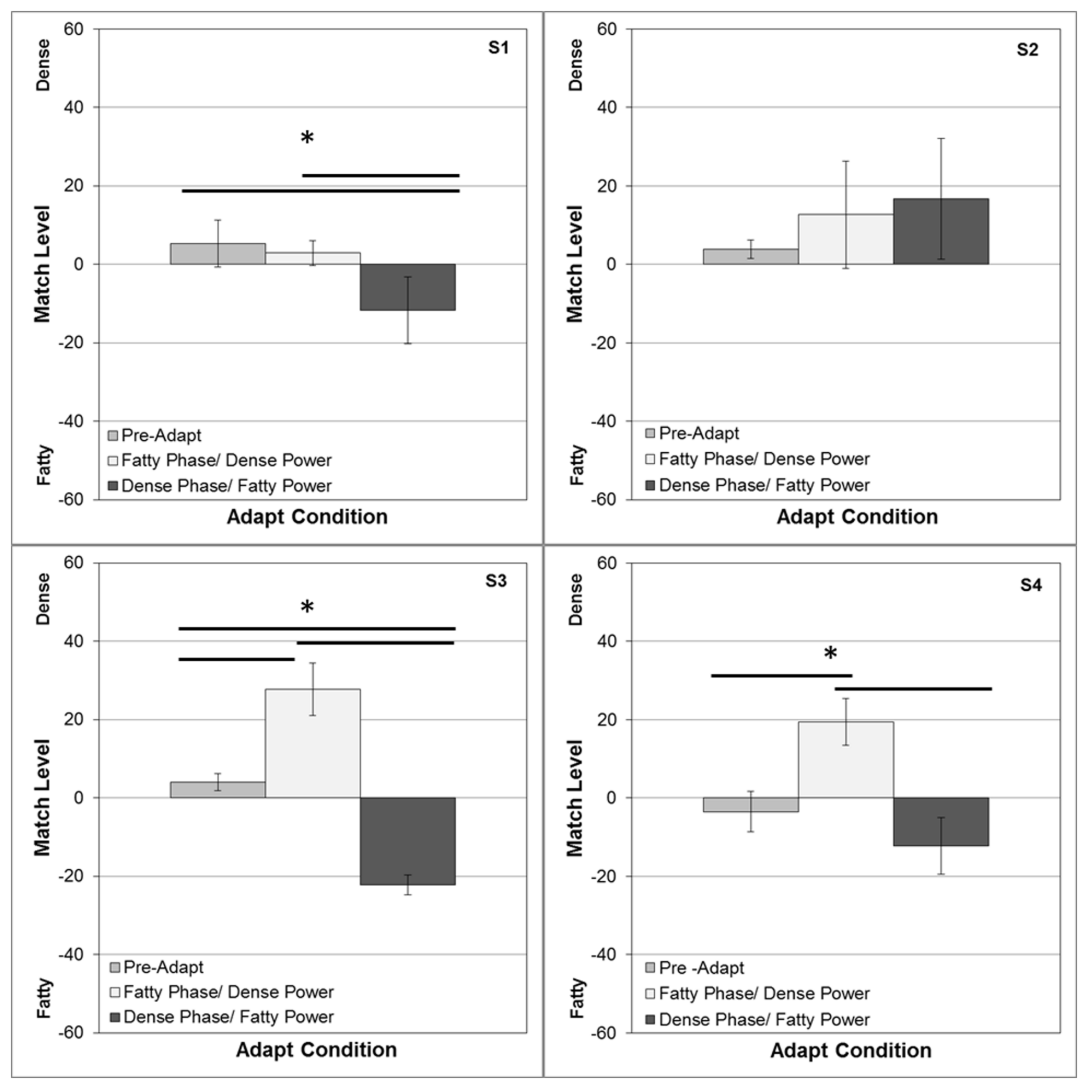
Aftereffects for the phase-swapped images. Aftereffects were tested as in Figure 4. Bars show the mean settings +1 standard error, when there were no prior adapting images (left), when the image with the fatty phase and dense power was on the left and the image with the dense phase and fatty power was on the right (middle), or when the positions were reversed (right). Horizontal lines indicate significant differences in the settings for the 3 conditions.

As a further test of the stimulus properties responsible for the measured aftereffects, we explored the specific role of the power and phase spectra of the images. The perceptual differences between most natural images are largely carried by the differences in phase spectra[27], though differences in the amplitude spectra can also strongly influence which image is perceived [28]. As noted in the introduction, medical images have steeper power spectra (with power decreasing with increasing spatial frequency roughly as p ~ f^-3^) than typical natural images (p ~ f^-2^) [5], and changes in the amplitude spectrum can be a powerful stimulus for spatial adaptation [21]. The slopes of the power spectra are similar for the fatty and dense images (which had average spectra of f^-2.87^ sd = .039 and f^-2.80^ sd = .145 respectively), but this reflects the spectrum averaged across all orientations, and images with the same slope but different anisotropies (e.g. with astigmatic blur) can also lead to strong and selective blur aftereffects [29].. We directly tested the relative influence of the amplitude and phase spectra on the adaptation by pitting them against each other. Figure 7 shows a pair of dense and fatty sections after swapping the phase spectra between the images while retaining the power spectrum of each image. The identity of each more closely follows the phase spectrum. We used these swapped images to similarly test whether the direction of the textural aftereffect followed the power or the phase spectrum. This experiment used the same procedures and images as in the double-adapt paradigm of Figure 4, except that the adapt image pairs were replaced with the hybrid images as in Figure 7. Consistent with the perceptual differences, the sign of the aftereffects remained tied to the phase spectrum for 3 observers (with no significant aftereffect either way for a fourth observer), again suggesting that it is primarily an adaptation to the textural attributes of the images (Figure 8). 

**Figure 9:**
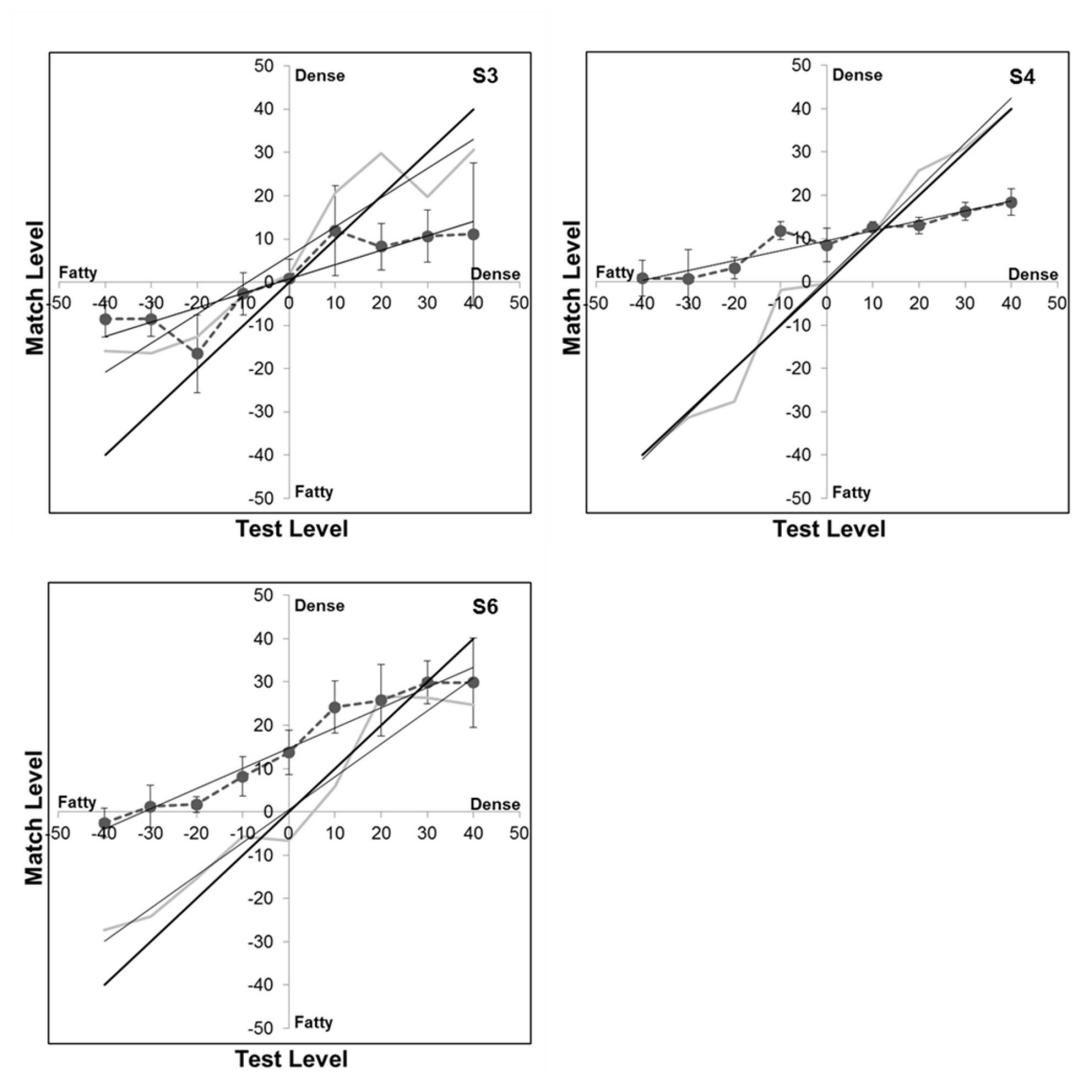
Changes in the appearance of the adapting image. Curves plot the matches made to different test levels before (gray line) or after (connected circles) adapting to the same level. Thin solid lines plot the linear regression lines. Thick solid line corresponds to the physical match. Panels plot the results for 3 observers.

In the preceding conditions the adapting images were always dense or fatty and thus at the extremes of the image array, while the probe images were at intermediate levels. The results were consistent with adaptation to either mammogram type causing test images to appear less like the image the observer was previously adapted to (though again these aftereffects were weaker for the dense adaptors). However, what happens to the appearance of the adapting image itself? Does its perceived texture change or do we simply become less sensitive to that texture? These questions have been of general importance to evaluate the nature of the perceptual changes resulting from adaptation [17,30]. For some dimensions (e.g. color) the stimulus appears weaker (e.g. less saturated) with prolonged viewing, consistent with a more global renormalization of the response so that the adapt stimulus appears more neutral (e.g. gray). For other attributes (e.g. size) the adapt level does not appear to change while both higher or lower levels appear biased away from the adapting level, consistent with a more local sensitivity change around the adapting level. To examine this, we modified the experiment so that the probe image now equaled the adapting image, and so that the effects were assessed not only for the original dense and fatty images but also for intermediate adapting levels. If the adapt image was not altered in appearance, then no aftereffects should have been observed under this condition. Instead, the matches were again strongly biased (Figure 9). Specifically, adaptation to denser images caused them to appear less dense, and adaptation to fatty images caused them to appear less fatty. In the results this is indicated by the reduction in the slope of the match settings for the different adapt levels (since these levels now appear more similar or intermediate), and these slope changes were significant (e.g. for the mean settings across observers F(1,50) = 5.015, p = .030). Notably, for two of the three observers, the appearance biases were not centered on the balanced average of the fatty and dense images but were instead biased toward moderately denser images (so that these moderately denser images were thus closer to the “neutral” point for the arbitrary texture dimension defining the stimulus array). This is again consistent with a weaker aftereffect for the dense images since by this criterion they differed less from the neutral stimulus and thus had a lower effective “contrast” along the dimension.

## Discussion

In summary, we have shown that brief exposures to different categories of mammogram images can lead to robust aftereffects in the appearance of the images. This has both general implications for adaptation and texture perception, and specific implications for the potential influence of adaptation on the visual perception of medical images. We consider these in turn.

As noted at the outset, the visual system can selectively adapt to a wide array of image properties from simple features to high-level attributes, and these adjustments can lead to very salient changes in appearance. The fact that adaptation could occur for properties of medical images is thus not surprising. However, it is important as a further illustration that adaptation can be manifest for the types of images and visual tasks that at least some observers are routinely exposed to, and thus as an example of the pervasive influence of adaptation on our perception [13]. Our results show that strong aftereffects can be induced by the differences that distinguish fatty vs. dense images, and suggest that these reflect the textural differences between the two image classes. Specifically, the aftereffects could not be accounted for by differences in contrast (which was nominally equated in the images) or power spectra (which were similar across the images and which did not predict the aftereffects; Figure 8). Instead, they followed the phase spectrum of the images, which also predicted the images’ appearance. Moreover, similar aftereffects occurred when observers were adapted to fatty or dense exemplars that were not the same images used in the test array (Figure 6). This indicates that the aftereffects could be induced by the spatial structure defining the dense or fatty categories separately from the identity of a specific image. 

Aftereffects on the appearance of visual texture have been demonstrated previously. For example, the perceived density of a dot texture can be biased by prior adaptation to a field with sparse or cluttered elements [31,32]. These density aftereffects occur for artificial and naturalistic textures, and as we found cannot be accounted for by differences in power spectra [33]. A recent study also found adaptation to the “regularity” of textures (e.g. arrays of elements with uniform or random spacing)[34]. Similarly, surface properties such as whether a material appears glossy or matte can be strongly biased by adaptation to the image statistics tied to these perceived attributes [35]. However, the effects of adaptation on texture perception are not well understood. One surprising finding with the images we studied is that the perceived texture of the adapting image itself changed with exposure – both fatty and dense images appeared less fatty or dense following adaptation (Figure 9).This is reminiscent of the perceptual changes that occur for attributes like color or facial configurations, which look less “saturated” with adaptation [17]. These have typically been accounted for by assuming that the underlying response changes occur within visual mechanisms that are broadly tuned for the stimulus dimension, and potentially as part of a norm-based code in which the stimulus is represented by how it differs from the norm [17,30]. By such accounts the aftereffects reflect a renormalization of the coding dimension so that the adapting level appears more neutral. Obviously, it is unclear what the actual visual coding dimensions are that underlie our ability to discriminate an arbitrary stimulus variation like fatty vs. dense images. However, whatever they are, they intriguingly behave as if they have a coding norm, and moreover adaptation can be used as a potential tool for defining this norm. In particular, the image level that does not change in appearance with adaptation is a plausible candidate for the neutral point in the continuum, since this is presumably the stimulus level to which the visual system is already adapted and thus “in balance” for [36]. In Figure 9, this corresponds to the point at which the pre- and post-adapt settings intersect. As noted, these are biased toward denser images (relative to the stimulus averages we created), and consistent with this, dense images also appeared to be less effective as adaptors. This predicts that image textures that are classified as dense might effectively be less “saturated” and thus possibly less visually salient than fatty textures, again because they are closer to the neutral point.

Before discussing potential implications of these aftereffects for the visual evaluation of medical images, several limitations of our study should be noted. First, as noted in the Introduction we did not test radiologists but instead untrained observers. There is no reason to think that observers are less susceptible to adapt to images that they have more familiarity with, and in fact the converse is possible. Yet as discussed below, the dynamics or form of the aftereffects could be affected by extensive experience. Second, our stimuli were hybrids formed by blending actual images, and may not suitably capture the properties of images that are rated as intermediate on the BI-RADS scale. Also, because our observers could not use this scale, it remains to be seen whether the adaptation could be strong enough to significantly alter the classification of the image. Finally, the adapting procedure we used was designed to generate strong and stable states of adaptation for specific properties of the images, and is very different from the sequence of stimulus exposures that might occur during a radiological reading. Thus the degree to which adaptation might impact performance in an actual screening remains to be explored.

Nevertheless, our results show that observers can strongly adapt to characteristics that distinguish mammogram images, and thus suggest that pattern-selective adaptation is potentially a significant but previously unrecognized factor affecting the perception and interpretation of medical images. In the simplest scenario, the aftereffects predict that the current image may tend to look less like the images viewed previously, giving rise to potential order effects in how images are evaluated. Moreover, our findings also point to the potential for changes in the perception of the current image itself depending on the duration of the inspection. Again, whether these turn out to play a measurable role in actual radiological settings is a question for future research, but could be explored by varying the sequence and timing of the image sets. 

There may also be positive consequences of adaptation. The functional benefits of pattern adaptation have remained difficult to demonstrate [13-15]. One prevalent account is that adaptation might enhance discrimination of stimuli similar to the adaptor by centering neural responses at the adapting level, though evidence for this is limited. Another account is that it allows the visual system to predict and thus discount the expected properties of the environment so that neural and perceptual resources can be devoted to unexpected properties. Interestingly, this in some ways mirrors the task confronting the radiologist, who must search the image to identify anomalous features. There is in fact some evidence that adaptation can enhance the salience of statistical outliers in stimulus distributions [37,38], and thus could in theory aid the radiologist by making rarer features in the image more perceptually conspicuous.

Finally, the context of medical image perception provides unique opportunities for exploring a number of unanswered questions about the nature of visual adaptation. Again, this is because the images themselves have “unnatural” but well characterized statistics and because radiologists have very extensive exposure to them. This allows for examining unresolved issues such how adaptation interacts with other experience-dependent processes such as perceptual learning [39] or visual expertise, and how the processes of adaptation operate over much longer timescales than are normally possible in the lab. Recent studies have suggested that the dynamics of adaptation extend over multiple durations [40-45], and that even the form of the response changes might vary with the length of exposure [46]. A further possibility is that the visual system might exhibit context-dependent adaptation so that it can store and rapidly engage different response states appropriate for different environments [47]. That is, an observer like a radiologist may be able to perceptually adjust more quickly to a context they have previously encountered. Because radiologists have had very long-term exposure to types of images that untrained observers rarely see, measurements of the nature of their own adaptation to medical images could also help reveal functional consequences of adaptation that are generally hidden in typical experimental paradigms or populations, either because the performance benefits require long adapting periods to emerge or because they are hidden in studies of natural images because observers are already expert (and thus there is no “untrained” group for comparison) [48]. 

## Supporting Information

Video S1 **Adaptation demo.** A demonstration of the basic textural aftereffects induced by adaptation to dense and fatty images.
(MPG)
